# Extract from *Syringa vulgaris* L. Flowers—A Special Emphasis on Its Biological Activity: Evaluation of Antioxidant Properties and Modulation of Coagulation Process in Human Plasma In Vitro

**DOI:** 10.3390/nu18071022

**Published:** 2026-03-24

**Authors:** Natalia Sławińska, Jerzy Żuchowski, Barbara Moniuszko-Szajwaj, Bartosz Skalski, Beata Olas

**Affiliations:** 1 Department of General Biochemistry, Faculty of Biology and Environmental Protection, University of Lodz, 90-236 Łódź, Poland; natalia.slawinska@biol.uni.lodz.pl; 2 Department of Phytochemistry, Institute of Soil Science and Plant Cultivation—State Research Institute, Czartoryskich 8, 24-100 Puławy, Poland; jzuchowski@iung.pulawy.pl (J.Ż.); bszajwaj@iung.pulawy.pl (B.M.-S.); 3 Department of Plant Physiology and Biochemistry, Faculty of Biology and Environmental Protection, University of Łódź, 90-237 Łódź, Poland; bartoszbartosz.1992@o2.pl

**Keywords:** coagulation, oxidative stress, plasma, *S. vulgaris* flowers

## Abstract

**Background/Objectives:** *Syringa vulgaris* L. (common lilac) is one of the most popular ornamental plant species. Through the ages, many parts of *S. vulgaris*, including fruits, flowers, leaves, and branches, have been used in folk medicine due to their beneficial biological activity. Lilac flowers are the basis of many supplements available on the market. Moreover, its petals and flowers are edible and are an aromatic ingredient in preserves and desserts. However, the data about the antioxidant properties of various parts of *S. vulgaris* is limited only to the in vitro antioxidant capacity of the extracts—so far, the effect of *S. vulgaris* flower extract on the parameters of oxidative stress in biological materials, including plasma, has not been demonstrated. Therefore, the aim of our study was to investigate the protective effects of the extract from *S. vulgaris* L. flowers against oxidative stress in human plasma, and its influence on the coagulation process in vitro. **Methods:** We measured the levels of three parameters of oxidative stress in human plasma treated with H_2_O_2_/Fe^2+^ (the donor of hydroxyl radicals): lipid peroxidation (based on the level of thiobarbituric acid reactive substances (TBARS)), protein carbonylation, and thiol oxidation. Ascorbic acid (vitamin C) was used as a reference antioxidant. In addition, we studied the effect of the extract on three coagulation parameters of human plasma-activated partial thromboplastin time (APTT), prothrombin time (PT), and thrombin time (TT). We also compared the biological properties of the extract from *S. vulgaris* flowers with the properties of a phenolic extract from *Taraxacum officinalis* (dandelion) flowers, as they have proven antioxidant activity in both in vitro and in vivo models and can modulate hemostasis in vitro. **Results:** Our UHPLC-HRMS analyses of *S. vulgaris* extract led to a tentative identification of 50 compounds, mainly phenolics and secoiridoids. For the first time, the present study demonstrated that the extract from *S. vulgaris* flowers (at the concentrations of 1–50 µg/mL) significantly reduced plasma lipid peroxidation and protein carbonylation induced by H_2_O_2_/Fe^2+^. Moreover, the concentrations of 1–25 µg/mL significantly reduced the oxidation of thiol groups in plasma treated with H_2_O_2_/Fe^2+^. The anticoagulant tests also demonstrated that *S. vulgaris* flowers extract, at physiologically relevant concentrations (1–50 µg/mL), did not affect blood clotting times in vitro, suggesting that it is hemostatically safe. **Conclusions:** Despite the differences in composition, the extracts from lilac flowers and dandelion flowers exhibited similar protective effects against oxidative damage to human plasma components. However, the extract from *S. vulgaris* flowers had a stronger inhibitory effect on lipid peroxidation than the extract from dandelion flowers.

## 1. Introduction

Various diseases, including cardiovascular diseases (CVDs) or cancers, are linked with the presence of oxidative stress. Oxidative stress can also modulate different elements of hemostasis, such as blood platelet function, or the coagulation process. On the other hand, research shows that phytochemicals can reduce the negative impact of oxidative stress on the organism and regulate hemostasis [1].

*Syringa vulgaris* L. (common lilac) is one of the most popular ornamental plant species. Through the ages, many parts of *S. vulgaris* (including fruits, flowers, leaves, and branches) have been used in folk medicine due to their antimicrobial, immunomodulating, and anti-inflammatory activities [2,3,4,5]. Lilac petals and flowers are edible—they can be added to various preserves and desserts as an aromatic additive. Popular products include honey, syrups, vinegars, teas, and infusions. Lilac flowers can also be used as an edible decoration in cakes or salads. However, data on the antioxidant properties of various parts of *S. vulgaris* are limited to measurements of their in vitro antioxidant capacity with methods such as ferric-reducing antioxidant power (FRAP), 2,2-diphenyl-picrylhydrazyl (DPPH•), and cupric ion reducing antioxidant capacity (CUPRAC) [3]. So far, the effect of *S. vulgaris* flower extract on oxidative stress parameters in biological samples, including plasma, has not been demonstrated. Therefore, the aim of our study was to investigate the protective effects of the extract from *S. vulgaris* L. flowers against oxidative stress in human plasma, and its influence on the coagulation process in vitro. We measured the levels of three parameters of oxidative stress in human plasma treated with H_2_O_2_/Fe^2+^ (the donor of hydroxyl radicals): lipid peroxidation (based on the level of thiobarbituric acid reactive substances (TBARS)), protein carbonylation, and thiol oxidation. Ascorbic acid (vitamin C) was used as a reference compound. In addition, we studied the effect of the extract on three coagulation parameters of human plasma-activated partial thromboplastin time (APTT), prothrombin time (PT), and thrombin time (TT). Importantly, we used concentrations below 50 µg/mL, which are usually achievable through oral administration.

We also compared the biological properties of the extract from *S. vulgaris* flowers with the properties of a phenolic extract from *Taraxacum officinalis* (dandelion) flowers. Dandelion is non-toxic and edible, which has led to its use in the food industry worldwide; its flowers are used to produce herbal teas, syrup, or wine. Dandelion flower extracts can be added to various food products, including cakes, desserts, puddings, and jellies, as flavoring agents [6]. Our earlier experiments demonstrated that *T. officinalis* flower extract possesses antioxidant activity not only in in vitro model, but also in vivo (in Wistar rats). Moreover, studies show that dandelion extract can also modulate hemostasis [7,8].

## 2. Materials and Methods

### 2.1. Chemicals

Methanol (isocratic grade), formic acid (LC-MS grade), acetonitrile (LC-MS grade), ammonium formate (for LC-MS), hexane, n-butanol, 5,5′-dithio-bis-(2-nitrobenzoic acid) (DTNB), thiobarbituric acid (TBA), guanidine hydrochloride, hydrogen peroxide, and sodium dodecyl sulfate (SDS) were acquired from Merck (Darmstadt, Germany). Trichloroacetic acid (TCA), EDTA, NaCl, ethanol, and ethyl acetate were from POCH (Gliwice, Poland). All coagulation times reagents were acquired from Diagon (Budapest, Hungary). All other reagents were acquired from commercial suppliers, including POCH (Gliwice, Poland), Chempur (Piekary Śląskie, Poland), and Merck (Darmstadt, Germany).

### 2.2. Plant Material

Inflorescences of the common lilac (*Syringa vulgaris* L.) were collected on 1 June 2021 in the village Łęka, Lublin Voivodeship, Poland (51°27′ N, 21°54′ E). Flowers were manually separated from the stalks, frozen at −18 °C, and freeze-dried (Gamma 2-16 LSC, Christ, Osterode am Harz, Germany). The dry flowers were powdered using a laboratory mill (ZM200, Retsch, Haan, Germany). A voucher specimen labeled 1/06/2021 has been deposited at the Institute of Soil Science and Plant Cultivation (Puławy, Poland).

Dandelion flowers were obtained from a farm in Rzeszów, Poland (50.114175 N, 21.911738 E) at the time of dandelion flowering (29 April 2015). Subsequently, plant material was freeze-dried, powdered, and used for extraction.

### 2.3. Preparation of S. vulgaris Extract

A portion of the milled flowers (80 g) was extracted overnight, with 2 L of 80% methanol (*v*/*v*), at room temperature; the extraction was assisted by ultrasonication (15 min). The extract was filtered, and the residual plant material was subjected to two further extractions (15 min, with ultrasonication) with new portions (1.5 L) of the same solvent. The obtained extracts were pooled, and methanol was removed by rotary-evaporation. After freezing, the preparation was freeze-dried (Gamma 2-16 LSC, Christ), to yield 45.42 g of the crude lilac flower extract. In the next step, sugars and other highly polar compounds were removed by solid phase extraction. The extract (45.42 g) was dissolved in 1% methanol with 0.1% formic acid (in MilliQ water; *v*/*v*), and loaded onto a C18 column (10 × 5 cm; Cosmosil 140C18-Prep, 140 μm). Next, the column was washed with the same solvent, in order to remove highly polar compounds, while phenolics and other specialized metabolites were eluted with 85% methanol (*v*/*v*). The eluate was concentrated in a rotary evaporator, frozen, and freeze-dried, yielding 15.12 g of the purified extract.

### 2.4. Phytochemical Analysis of the Plant Extracts

The composition of the lilac extract was analysed by UHPLC-HRMS, using a Thermo UltiMate 3000RS (Thermo Fischer Scientific, Waltham, MA, USA) chromatographic system, coupled with a Bruker Impact II Q-TOF mass spectrometer (Bruker Daltonics GmbH, Bremen, Germany). The extract was chromatographed on an ACQUITY UPLC HSS C18 column (2.1 × 100 mm, 1.8 µm; Waters, Milford, MA, USA), at 40 °C; the injection volume was 2.5 µL. The mobile phase A was 0.1% formic acid and 10 mM ammonium formate in MilliQ water, the mobile phase B was 0.1 formic acid in acetonitrile. The flow rate was 0.400 mL min^−1^, the following elution program was applied: 0.0–1.0 min: 2% B, 1–27.0 min: a concave gradient 2–40% B (the Chromeleon gradient No. 6), 27.5–30.0: 90% B, 30.5–32.0 min: 2% B. MS analyses were performed in negative and positive ion mode. The following settings were applied in negative ion mode: capillary voltage was 3000 V, dry gas temperature was 200 °C, nebulizer pressure was 0.7 Bar; collision RF was 700 Vpp; transfer time was 90 μs; prepulse storage time was 10 μs. Depending on the *m*/*z* values of fragmented ions, collision energy was set automatically in the range from 7 to 105 eV. The scanning range was *m*/*z* 80–1500. The mass spectrometer settings for positive ion mode: capillary voltage was 4500 V, dry gas temperature was 200 °C, nebulizer pressure was 0.7 Bar; collision RF was 700 Vpp; transfer time was 87.5 μs; prepulse storage time was 10 μs. Collision energy was set automatically in the range from 9 to 50 eV. The scanning range was *m*/*z* 80–1500. Constituents of the extracts were tentatively identified on the basis of their HRMS spectra, calculated formulas, and literature data.

To enable the evaluation of the relative content of phenolic compounds, including aromatic-conjugated secoiridoids, and provide information about their UV spectra, the extract was additionally analysed using an ACQUITY Premier^®^ UHPLC system (Waters Corporation, Milford, MA, USA), equipped with a PDA detector, and coupled with a Xevo TQ-XS triple quadrupole mass spectrometer (Waters Corporation, Milford, MA, USA). The sample was separated on an ACQUITY UPLC HSS C18 column (2.1 × 100 mm, 1.8 µm; Waters), at 40 °C; the injection volume was 1 µL. The mobile phase A was 0.1% formic acid in MilliQ water, the mobile phase B was 0.1 formic acid in acetonitrile. The flow rate was 0.400 mL min^−1^, the following elution program was applied: 0.0–1.0 min: 2% B, 1–27.0 min: a concave gradient 2–40% B (the MassLynx gradient No. 7), 27.2–29.0: 90% B, 29.2–32.0 min: 2% B. The mass spectrometer was operated in negative and positive ion scanning modes (range *m*/*z* 100–1500). The following settings were used in negative mode: capillary voltage 2.80 kV, cone voltage 35 V, source temperature 150 °C, desolvation temperature 600 °C, cone gas (N_2_) flow 150 L h^−1^, desolvation gas (N_2_) flow 1000 L h^−1^. In positive ion mode, the capillary voltage was 3.0 kV and the cone voltage was 40 V.

By applying LC-PDA-MS/MS technique 52 phenolic compounds, including 25 phenolic acids and their derivatives and 27 flavonoids, were characterized and tentatively identified in dandelion flowers. The chromatographic analyses showed that flavonoids were dominant compounds in the extract from dandelion flowers and their total amount equaled 633.71 mg/g of dry weight. luteolin was present in the highest amount among different flavonoids. This extract did not contain phenolic acids. More details about its isolation and phytochemical characteristics were previously described by Jędrejek et al. [7].

### 2.5. Preparation of Stock Solutions of the Plant Extracts and Ascorbic Acid for Bioassays

The extracts were dissolved in 50% DMSO and the pure compound-ascorbic acid in 75% DMSO. The final concentration of DMSO in the tested human plasma was below 0.5% (*v*/*v*) (for the extract) and 0.75% (*v*/*v*) (for the pure compound). DMSO is a universal solvent for many phytochemicals that does not affect the antioxidant or hemostatic properties of plasma at tested concentrations.

### 2.6. Blood Samples

Blood samples were collected from “Diagnostyka” blood collection center located at Brzechwy 7A Street (Łodź, Poland). An informed consent form was signed by all donors one day prior to blood collection. The donors did not smoke, take medication, or drink alcohol for two weeks before the experiment. Blood was anticoagulated with CPDA (citrate/phosphate/dextrose/adenine; 8.5:1; *v*/*v*; blood/CPDA). Full blood was centrifuged at 2800× *g* for 20 min at room temperature to obtain plasma. The research was conducted according to the guidelines of the Helsinki Declaration for Human Research, with the approval of Bioethics Committee at the University of Łódź (2/KBBN-UŁ/III/2014).

### 2.7. Lipid Peroxidation Measurement

To measure lipid peroxidation, a method based on thiobarbituric acid-reactive substances (TBARS) was used. First, the extract (or ascorbic acid) was added to plasma together with an oxidative stress inducer (4.7 mM H_2_O_2_/3.8 mM Fe^2+^/2.5 mM EDTA). The samples were incubated for 30 min at 37 °C. The final concentrations of the extract were 1–50 μg/mL. The negative control sample contained 0.9% NaCl instead of the extract. Next, equal amounts of 15% TCA and 0.37% TBA (both reagents dissolved in 0.25 M HCl) were added to all the samples; afterwards, they were incubated at 100 °C 15 min. After a brief cooling period, the samples were centrifuged at 10,000× *g* for 15 min at 18 °C. The supernatant was collected and the absorbance at 535 nm was measured with a SPECTROstar Nano Microplate Reader (BMG LABTECH, Ortenberg, Germany) in triplicate. For the blank sample, equal amounts of 0.9% NaCl, 15% TCA, and 0.37% TBA were used. The concentration of TBARS was calculated with a molar extinction coefficient (ε = 156,000 M^−1^ cm^−1^) [9].

### 2.8. Protein Carbonylation Measurement

A method based on 2,4-dinitrophenylhydrazine (DNPH) was used to measure protein carbonylation; this method was described by Bartosz [9] and Levine et al. [10]. First, the extract (or ascorbic acid) was added to plasma together with an oxidative stress inducer (4.7 mM H_2_O_2_/3.8 mM Fe^2+^/2.5 mM EDTA). The samples were incubated for 30 min at 37 °C. The final concentrations of the extract were 1–50 μg/mL. The negative control sample contained 0.9% NaCl instead of the extract. After the incubation, 40% TCA was added to all samples (on ice). The samples were subsequently incubated for 5 min and centrifuged for 5 min at 2500 rpm and 4 °C. The supernatant was discarded; 10 mM DNPH in 2 M HCl was added to the remaining pellets. After 1 h incubation (at room temperature in the dark) 40% TCA was added on ice; the samples were once more incubated for 5 min and centrifuged (5 min, 2500 rpm, 4 °C). After discarding the supernatant, 1.5 mL of 1:1 ethanol/ethyl acetate was added to the pellets on ice. The samples were vortexed for 5 min and centrifuged for 5 min, at 2500 rpm and 4 °C. This procedure was repeated twice. After discarding the supernatant for the last time, 1 mL 6 M guanidine hydrochloride in 2 M HCl was added; the samples were vortexed until the pellets were dissolved. The dissolved samples were pipetted onto a 96-well plate (each sample in triplicate) and absorbance was measured with a SPECTROstar Nano Microplate Reader (BMG LABTECH, Ortenberg, Germany) at 280 and 375 nm. The levels of carbonyl groups were calculated with a molar extinction coefficient (ε = 22,000 M^−1^ cm^−1^) and expressed as nmol carbonyl groups/mg of plasma protein.

### 2.9. Thiol Group Oxidation Measurement

The levels of thiol groups were assessed with a method based on Ellman’s reagent (5,5′-dithio-bis-(2-nitrobenzoic acid), DTNB). First, the extract (or ascorbic acid) was added to plasma together with an oxidative stress inducer (4.7 mM H_2_O_2_/3.8 mM Fe^2+^/2.5 mM EDTA). The samples were incubated for 30 min at 37 °C. The final concentrations of the extract were 1–50 μg/mL. The negative control sample contained 0.9% NaCl instead of the extract. After the incubation, 20 μL of the samples were added to a 96-well plate in triplicate. 20 μL of 10% SDS in 10 mM phosphate buffer (pH 8) and 160 μL of 10 mM phosphate buffer (pH 8) were added to all samples. After brief mixing, a SPECTROstar Nano Microplate Reader (BMG LABTECH, Ortenberg, Germany) was used to measure the absorbance of the samples at 412 nm and 280 nm. Next, 16.6 μL of 10 mM DTNB in 10 mM phosphate buffer (pH 8) was added to all the samples (16.6 μL of 10 mM phosphate buffer (pH 8) was added to the blank sample instead of DTNB). The plate was covered with parafilm, mixed, and incubated at 37 °C for 1 h; after that time, the absorbance was read again. The levels of thiol groups were calculated with a molar extinction coefficient (ε = 13,600 M^−1^ cm^−1^) and expressed as nmol thiol groups/mg of plasma protein [9].

### 2.10. Measurement of Prothrombin Time

The extract was added to plasma at the final concentrations of 1–50 μg/mL and incubated for 30 min at 37 °C. The negative control sample contained 0.9% NaCl instead of the extract. All samples were measured in duplicate. 50 μL of the samples were added to coagulometer cuvettes and incubated for 2 min at 37 °C. Next 100 μL of Dia-PT reagent was added. The coagulation time was recorded with a K-3002 Optic Coagulometer (Kselmed, Grudziądz, Poland). This method was described earlier by Sławińska et al. [11].

### 2.11. Measurement of Thrombin Time

The extract was added to plasma at the final concentrations of 1–50 μg/mL and incubated for 30 min at 37 °C. The negative control sample contained 0.9% NaCl instead of the extract. All samples were measured in duplicate. 50 μL of the samples were added to coagulometer cuvettes and incubated for 1 min at 37 °C. Next, 100 μL of thrombin (final concentration—5 U/mL) was added. The coagulation time was recorded with a K-3002 Optic Coagulometer (Kselmed, Grudziądz, Poland). This method was described earlier by Sławińska et al. [11].

### 2.12. Measurement of Activated Partial Thromboplastin Time

The extract was added to plasma at the final concentrations of 1–50 μg/mL and incubated for 30 min at 37 °C. The negative control sample contained 0.9% NaCl instead of the extract. All samples were measured in duplicate. 50 μL of the samples were added to coagulometer cuvettes. Next, 50 μL of Dia-PTT reagent (pre-heated to 37 °C) was added and the samples were incubated for 3 min at 37 °C. Afterwards, 50 μL of Dia-CaCl_2_ reagent was added to the cuvettes. The coagulation time was recorded with a K-3002 Optic Coagulometer (Kselmed, Grudziądz, Poland). This method was described earlier by Sławińska et al. [11].

### 2.13. Statistical Analysis

Statistical analysis was performed using Statistica 10 (StatSoft 13.3, TIBCO Software Inc., Palo Alto, CA, USA). Data distribution was evaluated with Shapiro-Wilk test, while variance homogeneity was assessed with Levene’s test. If the data had normal distribution and homogenous variance, the differences between groups were analyzed with a one-way ANOVA with Tukey’s post-hoc test; otherwise, Kruskal-Wallis test was applied. The results are expressed as means ± SD. The results were considered significant at *p* < 0.05. Dixon’s Q-test was used to eliminate uncertain data.

## 3. Results

### 3.1. Chemical Characteristic of the Plant Extract

**Figure 1:**
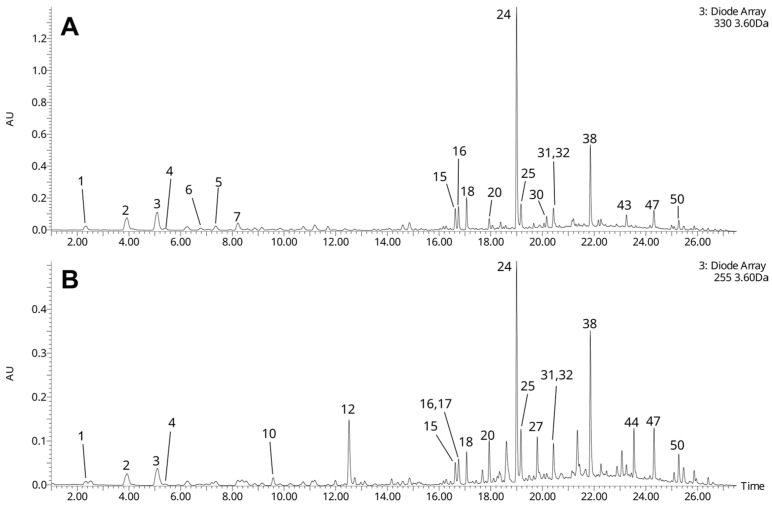
UHPLC-UV chromatograms of the lilac flower extract, registered at λ = 330 nm (**A**), and λ = 255 nm (**B**) (recorded using ACQUITY Premier^®^ UHPLC system). Numbers of the chromatographic peaks correspond to numbers of compounds in Table 1.

The UHPLC-HRMS analyses allowed for the tentative identification of 50 compounds, mainly phenolic compounds and secoiridoids (Table 1). Putative verbascoside (acteoside) seemed to be the dominant phenolic constituent of the extract (Figure 1). Other phenylethanoid glycosides were present in distinctly smaller amounts, mainly putative echinacoside, hydroxyacteosides, forsythoside B, isoverbascoside, as well as tyrosol hexoside, and hydroxytyrosol hexoside. Aromatic-conjugated secoiridoids constituted another important group of the lilac phenolics, which comprised putative oleoacteoside (apparently the second most abundant phenolic constituent of the extract), as well as putative oleonuezhenide, nuezhenide, hydroxyoleoacteoside, oleuropein and ligustroside (Table 1, Figure 1). Flavonoids were present in low number and small amounts: deoxyhexosides-hexosides of kaempferol and quercetin (Table 1, Figure 1). Other phenolics included putative syringin, putative lariciresinol hexoside (lignan), several caffeates and coumarates of a hexaric acid, and a number of unidentified larger derivatives of caffeic and coumaric acid. Most (with the exception of syringin) seemed to occur in small amounts (Table 1, Figure 1). As regards the identified non-phenolic compounds, they were represented by terpenoid-conjugated secoiridoids (putative 2″-epi-frameroside), simple secoiridoids (putative 11-methyloleoside), and monoterpenoid glycosides. The lilac flower monoterpenoid glycosides were tentatively identified as lilac alcohol (or its isomers) hexosides, and lilac alcohol hexoside malonates. Some unidentified compounds were also detected (Table 1).

### 3.2. Biomarkers of Oxidative Stress in Plasma

**Figure 2:**
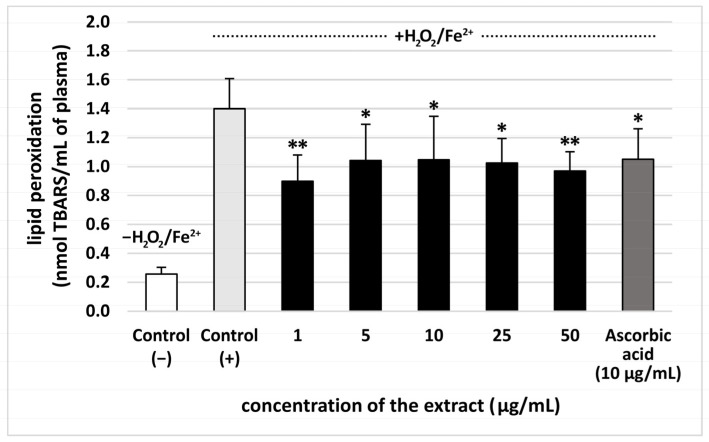
Effect of the extract from *S. vulgaris* flowers (1–50 µg/mL) and ascorbic acid (10 µg/mL) on thiobarbituric acid reactive substances (TBARS) levels in human plasma treated with Fe^2+^/H_2_O_2_ (an oxidative stress inducer) (*n* = 7). Control (+) and test samples (1–50 μg/mL) were incubated for 30 min at 37 °C, with Fe^2+^/H_2_O_2_. The data are expressed as means ± SD. The results were considered significant at *p* < 0.05 (* *p* < 0.05, ** *p* < 0.01). The difference between control (−) and control (+) was statistically significant (*p* < 0.001).

**Figure 3:**
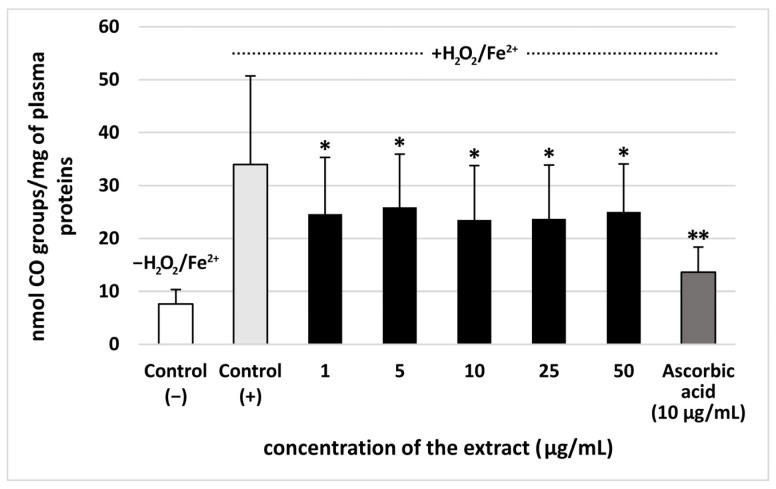
The effect of the extract from *S. vulgaris* flowers (1–50 µg/mL) and ascorbic acid (10 µg/mL) on carbonyl (CO) groups level in human plasma treated with Fe^2+^/H_2_O_2_ (an oxidative stress inducer) (*n* = 7). Control (+) and test samples (1–50 μg/mL) were incubated (30 min, 37 °C) with Fe^2+^/H_2_O_2_. The data are expressed as means ± SD. The results were considered significant at *p* < 0.05 (* *p* < 0.05; ** *p* < 0.01). The difference between control (−) and control (+) was statistically significant (*p* < 0.001).

**Figure 4:**
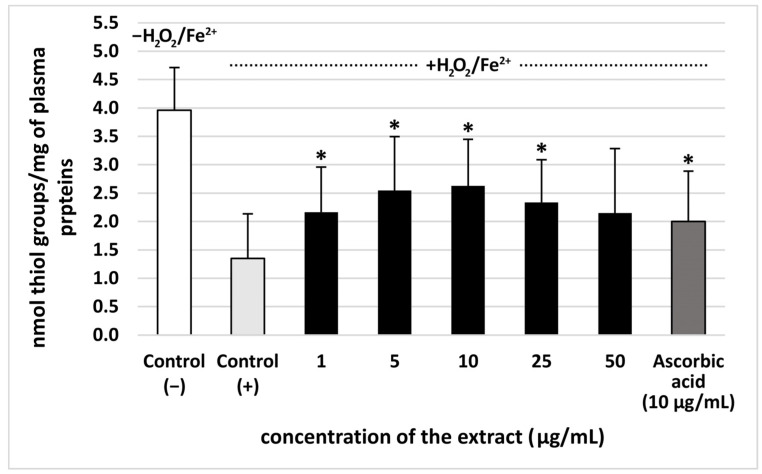
The effect of the extract from *S. vulgaris* flowers (1–50 µg/mL) and ascorbic acid (10 µg/mL) on thiol groups levels in human plasma treated with Fe^2+^/H_2_O_2_ (an oxidative stress inducer) (*n* = 7). Control (+) and test samples (1–50 μg/mL) were incubated (30 min, 37 °C) with Fe^2+^/H_2_O_2_. The data are expressed as means ± SD. The results were considered significant at *p* < 0.05 (* *p* < 0.05). The difference between control (−) and control (+) was statistically significant (*p* < 0.001).

The extract from *S. vulgaris* flowers (at all used concentrations: 1–50 µg/mL) significantly reduced plasma lipid peroxidation induced by H_2_O_2_/Fe^2+^, however, this effect was not dose-dependent. The % of inhibition observed for the extract at the highest concentration–50 µg/mL—was approximately 35% (Figure 2). Moreover, all used concentrations (1–50 µg/mL) of the extract from *S. vulgaris* flowers decreased plasma protein carbonylation stimulated by H_2_O_2_/Fe^2+^ (the % of inhibition was about 35%) (Figure 3). The highest concentration of the extract (50 μg/mL) did not change the level of thiol groups in plasma treated with H_2_O_2_/Fe^2+^ (Figure 4). However, the lower concentrations of the extract from *S. vulgaris* flowers (1–25 µg/mL) significantly reduced thiol groups oxidation (Figure 4).

The effects of the extract from *S. vulgaris* flowers (10 µg/mL) on oxidative stress were compared with the extract from *T. officinalis* flowers (10 µg/mL) in Table 2. The dandelion extract, which was used as a positive control (10 µg/mL), demonstrated antioxidant properties, but the extract from *S. vulgaris* flowers had stronger inhibitory effect on lipid peroxidation. On the other hand, the % of inhibition of protein carbonylation for two tested extracts (from *S. vulgaris* flowers and *T. officinalis* flowers) was very similar (Table 2).

### 3.3. Coagulation Times (PT, TT, and APTT)

**Figure 5:**
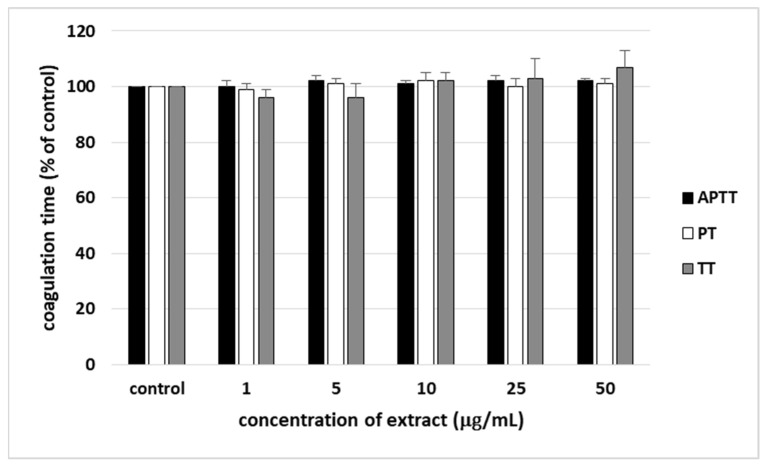
The effect of the extract from *S. vulgaris* flowers (1–50 µg/mL) on the hemostatic parameters of human plasma: APTT, PT, and TT (*n* = 6). In the graphs, the coagulation time is expressed as a percentage of the control sample (plasma without the tested extract). The data are expressed as means ± SDs. The results were considered significant at *p* < 0.05.

None of used concentrations of the extract from *S. vulgaris* flowers (1–25 µg/mL) significantly impacted the coagulation times measured in human plasma (Figure 5).

## 4. Discussion

The investigated lilac flower extract contained a broad array of specialized metabolites, belonging to different classes, mainly phenolic compounds (including phenylethanoid glycosides), and secoiridoids (Table 1, Figure 1). The presence of secoiridoids (frequently conjugated with a phenolic moiety) and phenylethanoid glycosides is a characteristic feature of plants from the *Oleaceae* family [12,13]. As shown by our UHPLC-MS analyses, the dominant phenolic constituent (also including aromatic-conjugated secoiridoids) of the extract from *S. vulgaris* flowers was putative verbascoside. Other phenylethanoid glycosides, such as putative hydroxyacteosides, echinacoside, forsythoide B, isoverbascoside, as well as hexosides of hydroxytyrosol and tyrosol were also present, but in much lower quantities. It seems that previous phytochemical research on *S. vulgaris* was focused mainly on the bark and leaves of the plant, while publications describing the flowers were scarce. Nevertheless, the above-mentioned compounds were reported in two earlier publications on the phytochemical composition of lilac flowers [5,14], while the presence of verbascoside and echinacoside was additionally confirmed by Hanganu et al. [3]. In addition, Toth et al. [14] also showed that the methanol extract from lilac flowers contained very high amounts of verbascoside, which seems to confirm our results. Apart from phenyetanoid glycosides, the lilac flower extract also contained three flavonoids, 3-*O*-hexosides-deoxyhexosides of quercetin and kaempferol. Two of them were most probably rutin (quercetin 3-*O*-rutinoside), and kaempferol 3-*O*-rutinose, as these compounds were previously isolated from *S. vulgaris* flowers [15]. Similar flavonoids were also detected in lilac flower extracts by other research teams [5,14]. Several caffeates and coumarates of a hexaric acid were also detected, which are most probably different caffeoylglucaric and *p*-coumaroylglucaric acids [2]. Such compounds were also detected in lilac flower extracts by LC-MS [5,14], as well as other *Oleacea* species, *Ligustrum vulgare* and *Fraxinus excelsior* [16,17]. Our lilac flower extract was also shown to contain significant amounts of putative syringin and trace quantities of putative lariciresinol hexoside. In the case of syringin, literature data are ambiguous. While *S. vulgaris* bark is a rich source of this substance [14,18,19], Tóth et al. [14] and Woźniak et al. [5] found only traces, or no syringin in the flower extracts. In contrast, Hanganu et al. [3] detected significant quantities of the compound in lilac flowers. It is quite possible that the content of syringin in *S. vulgaris* flowers may depend on the flower maturity state (the collection date), pedoclimatic conditions, and other factors. The detected lignan glycoside, putative lariciresinol hexoside is most probably (+)-lariciresinol 4-*O*-glucopyranoside, previously found in the bark of *S. vulgaris* [18,19]. Secoiridoids constitute another important group of the lilac specialized metabolites. Most of them are aromatic-conjugated secoiridoids, which may be regarded also as phenolic compounds. In the currently described extract, these compounds included oleoacteoside, oleonuezhenide (major compounds), as well as hydroxyoleoacteoside, nuezhenide, oleuropein and ligustroside. Simple secoiridoids (putative 11-methyloleoside), and terpenoid-conjugated secoiridoids (putative 2″-epi-frameroside) were also identified. All these compounds were previously detected in extracts from flowers of *S. vulgaris* [5,14]. In addition, several monoterpenoid hexosides, and putative malonylated monotorpenoid hexosides. The monoterpenoid hexosides are probably glucosides of lilac alcohols. Lilac alcohols (8 diastereoisomers) are among characteristic constituents of the lilac essential oil, and (βR,2R,5S)-lilac alcohol *β*-glucopyranoside was isolated from the flowers [2]. The tentatively-identified malonylated monoterponoid hexosides most probably have not been described before.

Our study is the first work devoted to a comprehensive assessment of biological effect of lilac flowers extract, employing in vitro experimental system related to the human plasma and blood physiology, which includes the coagulation process. The present study brings originality by offering, for the first time, the proof of the antioxidant activity of *S. vulgaris* flowers extract by using three assays—the level of TBARS (the final lipid peroxidation products), thiol groups, and carbonyl groups—in human plasma treated with H_2_O_2_/Fe^2+^ (the donor of •OH—one of the most aggressive reactive oxygen species generated in human). In addition, the inhibition of oxidative stress (especially lipid peroxidation and thiol oxidation) stimulated by H_2_O_2_/Fe^2+^ was comparable to or higher than the action of vitamin C (which is a potent water-soluble antioxidant) at 10 µg/mL.

Various secondary metabolites, including phenolic compounds, exhibit antioxidant activity within a defined therapeutic window, representing the exposure range in which they reduce oxidative stress without triggering prooxidant effects. At lower, physiologically compatible levels, they contribute to free radical scavenging and redox balance, as demonstrated in studies showing their role in mitigating oxidative stress. However, as exposure increases beyond the optimal range, different phenolic compounds begin to exhibit prooxidant effects. This dose-dependent duality underscores the need for careful characterization of safety margins when evaluating phenolic compounds as potential antioxidant agents [20]. Here, the tested extract from *S. vulgaris* flowers had the best antioxidant activity at the concentrations of 5 and 10 μg/mL, while the activity of 50 μg/mL extract was not significant, when we measured its effect on the level of thiol groups in plasma treated with H_2_O_2_/Fe^2+^. This indicates that *S. vulgaris* flower extract is more effective at preventing thiol groups oxidation at lower (5–10 μg/mL) concentrations.

The antioxidant mechanisms of lilac flower extract observed in human plasma might include scavenging oxidants (H_2_O_2_ and H_2_O_2_/Fe^2+^-derived •OH radicals). Our results are consistent with previous studies demonstrating the role of bioactive compounds from lilac flowers in protecting against oxidative stress. The antioxidant activity of lilac flowers (IC_50_ = 65.25 µg/mL) was noted by Tóth et al. [14] using the DPPH (2,2-diphenyl-1-picrylhydrazyl) bleaching assay. Results of Hanganu et al. [3] also demonstrated that the extract from *S. vulgaris* flowers has antioxidant potential, which was measured by three separate methods: DPPH, Ferric Reducing Antioxidant Power (FRAP), and Cupric Ion Reducing Antioxidant Capacity (CUPRAC) assay. Gąsecka et al. [21] noted that the scavenging capacity of the extract from lilac flowers is correlated with the total content of phenolic compounds, including flavonoids. These findings on the antioxidant potential of lilac flowers support the recommendation that *S. vulgaris* can be used as a supplement in managing atherosclerosis, as oxidative stress (particularly lipid peroxidation) is a key hallmark of this disease [22]. There are known supplements based on lilac flowers (e.g., Liliac PlantExtract produced in Romania) that are recommended in maintaining the function of the cardiovascular system by enhancing the elasticity of blood vessels. In addition, lilac flowers are a source of dietary fiber, which has cardioprotective potential [23].

Dudek et al. [2] observed that lilac flowers are a good source hydroxycinnamoyl derivatives and a secoiridoid glycoside with anti-inflammatory properties. These properties were observed in human neutrophils (in vitro) at the concentration of 50 µM. In addition, Woźniak et al. [5] found that neoleuropein—an especially active secoiridoid—decreased the production of various cytokines in human neutrophils through the inhibition of mitogen-activated protein kinase (MAP) kinases phosphorylation [5].

For the first time, our study provides a comparative analysis of lilac and dandelion flowers, demonstrating that *S. vulgaris* flowers, like *T. officinale* flowers, have antioxidant potential. Our results showed that the extract from lilac flowers exhibits stronger antioxidant activity, particularly in preventing plasma lipid peroxidation, than the phenolic extract from dandelion flowers. It seems probable that the differences in the chemical profiles of both extracts can explain the stronger action of the lilac extract, which contains not only various phenolic compounds, but also secoiroidoids—a diverse class of bioactive cyclopentane monoterpenoid derivatives formed by the cleavage of the cyclopentane ring, commonly found in plants of the *Oleaceae* family, including olives. Studies have found that secoiroidoids exhibit strong antioxidant, anti-inflammatory, neuroprotective, and anti-cancer properties. 

It is known that oxidative stress can affect various elements of hemostasis, including the coagulation cascade, which is a complex process [24,25,26,27]. For example, the results of Wang et al. [27] demonstrated that the cross-linking, branching, and height distribution of formed fibrin is influenced by the oxidative stress of fibrinogen induced by H_2_O_2_. On the other hand, this process can be modulated by various plant extracts. Both phenolic compounds and other low molecular secondary metabolites may modulate not only the coagulation process, but also fibrinolysis and blood platelet activation [1,28]. For example, the anticoagulant effect of *Rheum rhaponticum* and *Rheum rhabarbarum* extracts is partly attributed to the inhibition of the coagulation factor Xa and thrombin activities [29].

Evaluation of the clotting times (PT, TT, and APTT) is a simple and rapid screening test that enables the assessment of the effects of plant extracts on coagulation. In the present study, the effects of lilac flower extract on these three coagulation times were measured in human plasma; the anticoagulant tests demonstrated that *S. vulgaris* flowers extract, at physiologically relevant concentrations (1–50 µg/mL), did not affect blood clotting times in vitro, suggesting that it is hemostatically safe.

Biological properties of the tested plant extracts (including their effects on oxidative stress stimulated by H_2_O_2_/Fe^2+^ and coagulation process) were examined in the concentration range of 1–50 µg/mL, which was chosen based on our previous study [7] and literature data, indicating that the physiologically achievable concentrations of phenolic compounds in plasma are mostly at levels ranging from nanomoles to a few micromoles per liter [29]. Although no data on secroiridoid bioavailability after *S. vulgaris* extract intake is available, some information on the concentrations of these compounds from other plants in plasma can be found in literature. Secoiridoides have low to moderate oral bioavailability. Despite this, their plasma concentration peaks quickly—after about 1 h in humans [23].

## 5. Conclusions

In conclusion, this study is the first to comprehensively evaluate *S. vulgaris* flowers extract, combining the physiological characterization with the assessment of its antioxidant properties. Using UHPLC-HRMS, 50 compounds, mainly phenolic compounds and secoiridoids were identified. The examined extract from lilac flowers displayed the ability to inhibit oxidative stress induced by H_2_O_2_/Fe^2+^. Lilac flowers extract provided a significant protective effect against the oxidation of plasma lipids and proteins, at the levels achievable for phenolic compounds in plasma after oral supplementation, and with the action comparable to vitamin C and the extract from dandelion flowers. Secoiridoids, which are found in *S. vulgaris* flowers, could also show antioxidant potential, but this data is available only in vitro, for secoiridoids isolated from other plant sources [30,31,32]. Therefore, their beneficial biological properties need to be further explored in vivo. In addition, according to recent studies, these compounds have also demonstrated therapeutic potential in various CVDs [31].

The antioxidant properties, including the protection of molecules from oxidative damage, might thus contribute to the beneficial effects of lilac flower remedies used in the prophylaxis and treatment of CVDs. However, further research is required to deepen the understanding of the molecular mechanisms of action of *S. vulgaris* flowers extract, and verify its antioxidant effects in vivo.

## Figures and Tables

**Table 1:** Tentative identification of constituents of the extract from flowers of *S. vulgaris*.

	tR	Type	*m/z*	Major Fragment Ions (*m/z*)	Error (ppm)	mSigma	Formula	Tentative Identification
1	1.85	[M-H]^−^	371.0625	209.0303 (100), 191.0211 (15), 179.0341 (8)	−1.4	24.7	C_15_H_16_O_11_	caffeoylhexaric acid/isomer
2	2.39	[M-H]^−^	371.0617	209.0305 (100), 191.0188 (14), 179.0337 (2)	0.8	2.2	C_15_H_16_O_11_	caffeoylhexaric acid/isomer
3	2.55	[M-H]^−^	371.0626	209.0305 (100), 191.0197 (13), 179.0393 (2)	−1.7	7.1	C_15_H_16_O_11_	caffeoylhexaric acid/isomer
4	2.71	[M-H]^−^	355.0673	209.0307 (59), 191.0176 (100), 147.0281 (27)	−0.7	1.8	C_15_H_16_O_10_	coumaroylhexaric acid/isomer
5	3.36	[M-H]^−^	371.0614	209.0308 (100), 191.0198 (13)	1.6	5.1	C_15_H_16_O_11_	caffeoylhexaric acid/isomer
6	3.80	[M-H]^−^	355.0665	355.0671 (4), 209.0303 (99), 191.0203 (100), 163.0392 (9)	1.6	4.8	C_15_H_16_O_10_	coumaroylhexaric acid/isomer
7	4.26	[M-H]^−^	355.0665	209.0300 (100), 191.0202 (300)	1.5	8.6	C_15_H_16_O_10_	coumaroylhexaric acid/isomer
8	4.62	[M+NH_4_]^+^	332.1343	332.1346 (100), 315.1084 (2), 279.0868 (4), 219.0651 (5), 180.0871 (11), 163.0607 (2), 153.0546 (4), 157.0499 (7), 127.0399 (3)	−1.1	11.0	C_14_H_18_O_8_	unidentified hexoside
9	4.78	[M-H]^−^	315.1091	315.1088 (100), 135.0435 (2)	−1.9	9.3	C_14_H_20_O_8_	hydroxytyrosol-Hex/isomer
		[M+NH_4_]^+^	334.1501	334.1501 (100), 317.1241 (29), 281.1022 (14), 263.0917 (18), 221.0811 (7), 155.0704 (24), 137.0598 (31)	−1.4	12.0		
10	6.32	[M+FA-H]^−^	345.1194	345.1184 (4), 299.1135 (100), 179.0567 (6)	−0.8	7.7	C_14_H_20_O_7_	tyrosol-Hex/isomer
11	8.20	[M-H]^−^	403.1253	371.0950 (11), 241.0721 (52), 223.0606 (49), 197.9821 (80), 179.0712 (83)	−1.7	23.3	C_17_H_24_O_11_	oleoside 11-methyl ester hexoside/isomer
12	8.20	[M+FA-H]^−^	417.1400	209.0820 (100)	−0.5	10.8	C_17_H_24_O_9_	syryngin/isomer
		[M+NH_4_]^+^	390.1762	211.0965 (30), 193.0858 (100), 180.0867 (6), 161.0598 (45)	−1.0	10.5		
13	8.45	[M+FA-H]^−^	315.1089	315.1081 (38), 269.1028 (100)	−1.3	13.2	C_13_H_18_O_6_	unidentified
		[M+NH_4_]^+^	288.1439	288.1442 (9), 271.1178 (28), 253.1059 (100), 235.0955 (25), 217.0858 (25), 180.0865 (20), 161.0444 (17), 145.0496 (19), 127.0400 (6)	0.9	5.1		
14	10.00	[M+FA-H]^−^	447.1504	401.1453 (100), 269.1029 69), 161.0447 (2)	1.0	10.0	C_18_H_26_O_10_	unidentified
15	12.23	[M-H]^−^	639.1925	639.1931 (100), 621.1823 (38), 529.1546 (8), 487.1446 (8), 477.1606 (5), 179.0353 (13), 161.0243 (28)	0.9	9.6	C_29_H_36_O_16_	hydroxyverbascoside/iosomer
16	12.39	[M-H]^−^	639.1915	639.1931 (100), 621.1829 (74), 529.1533 (6), 487.1461 912), 477.1601 (5), 459.1500 (13), 179.0349 (19), 161.0242 (38), 151.0384 (6), 133.0282 (12)	2.5	24.8	C_29_H_36_O_16_	hydroxyverbascoside/isomer
17	12.44	[M+FA-H]^−^	461.1662	415.1604 (100), 311.0980 (4), 283.1187 (6), 251.0766 (7), 221.0669 (3), 191.0561 (7), 179.0561 (7), 161.0443 (1), 149.0449 (15), 131.0329 (2)	0.5	3.6	C_19_H_28_O_10_	unidentified
18	12.95	[M-H]^−^	785.2503	785.2514 (100), 623.2197 (24), 179.0349 (1), 161.0235 (26), 133.0275 (8)	0.8	22.9	C_35_H_46_O_20_	verbascoside-Hex (echinacoside)/isomer
		[M+NH_4_]^+^	804.2924	625.2123 (4), 479.1553 (13), 471.1503 (15), 325.0925 (100), 309.0975 (3), 163.0394 (49)	−0.4	3.2		
19	13.14	[M+NH_4_]^+^	350.2178	350.2174 (4), 333.1913 (10), 315.1807 (26), 297.1706 (15), 279.1599 (7), 163.0604 (4), 153.1278 (58), 135.1171 (100)	−1.3	11.8	C_16_H_28_O_7_	monoterpenoid hexoside (lilac alcohol hexoside ?)
20	13.87	[M-H]^−^	609.1459	609.1453 (90), 300.0270 (69), 271.0240 (100), 255.0295 (39), 243.0294 (21)	0.4	10.1	C_27_H_30_O_16_	quercetin-3-*O*-Hex-dHex (rutin ?)/isomer
21	14.48	[M-H]^−^	593.1506	593.1516 (50), 284.0326 (90), 255.0290 (100), 227.0345 (42)	1.0	12.9	C_27_H_30_O_15_	kaempferol-3-*O*-Hex-dHex/isomer
22	14.54	[M-H]^−^	521.2014	359.1496 (17), 329.1391 (100)	2.8	13.7	C_26_H_34_O_11_	lariciresinol-Hex
23	14.80	[M-H]^−^	755.2390	755.2398 (100), 593.2086 (18), 161.0239 (20), 133.0286 (8)	1.9	13.1	C_34_H_44_O_19_	forsythoside B/isomer
		[M+NH_4_]^+^	774.2824	774.2819 (14), 625.2130 (4), 479.1557 (11), 471.1508 (19), 457.1353 (1), 325.0929 (100), 309.0982 (4), 181.0505 (2), 163.0396 (54)	−1.1	10.2		
24	15.55	[M-H]^−^	623.1973	623.1974 (100), 461.1659 (10), 161.0239 (21), 133.0287 (7)	1.4	2.6	C_29_H_36_O_15_	verbascoside/isomer
25	15.71	[M+H]^+^	595.1657	595.1656 (3), 449.1081 (14), 287.0553 (100)	0.0	13.1	C_27_H_32_O_15_	kaempferol-3-*O*-Hex-dHex/isomer
26	16.66	[M+FA-H]^−^	377.1811	377.1815 (53), 331.1752 (100), 161.0450 (2)	1.5	1.4	C_16_H_28_O_7_	monoterpenoid hexoside (lilac alcohol hexoside ?)
27	16.82	[M+FA-H]^−^	731.2403	685.2343 (25), 523.1818 (100), 453.1396 (95), 421.1500 (52), 299.1135 32), 223.0608 (18), 153.0188 (4)	0.1	9.8	C_31_H_42_O_17_	nuezhenide/isomer
		[M+NH_4_]^+^	704.2764	704.2763 (27), 525.1973 (15), 507.1867 (51), 387.1291 (42), 369.1184 (55), 295.0815 (18), 193.0496 (47), 165.0547 (100), 151.0391 (26)	−0.5	10.3		
28	16.55	[M-H]^−^	623.1968	623.1974 (100), 461.1650 (10), 179.0338 (3), 161.0232 (19), 133.0283 (5)	2.2	8.7	C_29_H_36_O_15_	verbascoside isomer
29	17.28	[M+NH_4_]^+^	350.2173	333.1909 (36), 171.1379 (100), 153.1273 (5), 135.1167 (8)	0.2	7.3	C_16_H_28_O_7_	monoterpenoid hexoside (lilac alcohol hexoside ?)
30	17.28	[M-H]^−^	607.2025	607.2022 (100), 461.1658 (47), 443.1581 (2), 315.1075 (2), 163.0386 (3), 145.0285 (23), 117.0337 (7)	1.2	5.4	C_29_H_36_O_14_	deoxyverbascoside
31	17.95	[M+NH_4_]^+^	1044.355	1044.3541 (7), 847.2651 (5), 829.2542 (10), 677.2074 (14), 587.1760 (8), 505.1706 (14), 477.1394 (31), 371.1339 (21), 325.0920 (100), 225.0756 (24), 193.0495 (17), 165.0546 (56), 163.0390 (62)	0.4	18	C_46_H_58_O_26_	hydroxyoleoacteoside
32	18.08	[M+NH_4_]^+^	1190.4100	1190.4109 (14), 993.3212 (4), 831.2690 (12), 677.2068 (14), 589.1909 (4), 479.1545 (17), 371.1332 (15), 353.1228 (19), 325.0918 (100), 225.0755 (21), 193.0494 (13), 165.0544 (46), 163.0389 (56)	2.8	23.4	C_52_H_68_O_30_	oleoechinacoside/isomer
33	18.36	[M+NH_4_]^+^	436.2177	436.2175 (8), 419.1908 (68), 401.1803 (4), 383.1707 (1), 249.0604 (2), 231.0498 (6), 171.1378 (100), 153.1273 (8), 135.1166 (11)	0.0	4.5	C_19_H_30_O_10_	monoterpenoid hexoside-MaA/isomer
		[2M-H]^−^	835.3594	373.1853 (25), 331.1747 (100)	1.3	8.9		
34	18.92	[M+NH_4_]^+^	436.2178	436.2175 (10), 419.1911 (89), 401.1806 (4), 383.1700 (2), 249.0603 (2), 231.0498 (5), 171.1378 (100), 153.1272 (7), 135.1166 (10)	−0.2	13.0	C_19_H_30_O_10_	monoterpenoid hexoside-MaA/isomer
35	19.19	[M-H]^−^	539.1756	403.1248 (27), 377.1226 (69), 345.0974 (23), 307.0809 (100), 275.0913 (76), 223.0615 (24), 179.0561 (8), 149.0234 (8)	2.7	7.8	C_25_H_32_O_13_	oleuropein/isomer
		[M+NH_4_]^+^	558.2184	541.1920 (2), 379.1388 (54), 361.1282 (100), 347.1126 (16), 287.0916 (7), 225.0759 (8), 165.0547 (15), 137.0596 (69)	−0.4	5.3		
36	19.45	[M+NH_4_]^+^	436.2178	419.1912 (68), 401.1806 (2), 383.1700 (2), 339.1803 (2), 249.0604 (1), 231.0499 (3), 171.1378 (100), 153.1274 (5), 135.1166 (8)	−0.2	18.6	C_19_H_30_O_10_	monoterpenoid hexoside-MaA/isomer
37	19.77	[M-H]^−^	677.2074	633.2170 (100), 591.2060 (1), 487.1784 (5), 445.1683 (2), 163.0388 (3), 145.0279 (29), 117.0340 (10)	2.0	17.4	C_32_H_38_O_16_	coumaric acid derivative
		[M+NH_4_]^+^	696.2493	969.2494 (7), 679.2225 (1), 541.1550 (13), 533.1653 (20), 395.0972 (100), 377.0863 (4), 291.0862 (3), 165.0546 (4), 147.0440 (21)	0.7	1.0		
38	20.25	[M-H]^−^	1009.3158	847.2812 (10), 745.2316 (8), 665.2068 (13), 623.1964 (21), 461.1651 (14), 315.1078 (4), 297.0971 (2), 179.0343 (3), 161.0235 (49), 133.0282 (15)	3.6	3.0	C_46_H_58_O_25_	oleoacteoside/isomer
		[M+NH_4_]^+^	1028.3606	1028.3592 (13), 831.2699 (8), 695.2179 (4), 677.2072 (17), 507.1863 (6), 479.1549 (15), 371.1338 (15), 325.0921 (100), 275.0915 (15), 225.0758 (19), 193.0496 (14), 163.0391 (50), 151.0392 (9)	0.0	8.8		
39	20.72	[M+NH_4_]^+^	460.1820	460.1816 (58), 443.1552 (16), 425.1446 (6), 303.1234 (6), 266.0871 (10), 231.0501 (19), 195.1018 (100), 163.0757 (10)	−1.5	10.7	C_20_H_26_O_11_	unidentified
40	20.93	[M+NH_4_]^+^	1028.3595	1028.3591 (9), 831.2704 (6), 704.2258 (5), 685.2124 (74), 549.1604(57), 531.1497 (74), 307.0819 (35), 225.00758 (29), 193.0497 (33), 165.0458 (100), 163.0392 (97)	1.0	10.3	C_46_H_58_O_25_	oleoacteoside/isomer
		[M-H]^−^	1009.3170	1009.3166 (100), 873.2647 (11), 847.2780 (9), 745.2338 (13), 665.2057 (15), 623.1995 (14), 461.1639 (13), 403.1244 (4), 315.1065 (1), 297.1005 (4), 179.0343 (31), 161.0238 (66)	2.5	16.5		
41	21.46	[M-H]^−^	523.1812	361.1276 (42), 291.0865 (100), 259.0965 (23)	1.8	17.3	C_25_H_32_O_12_	ligstroside/isomer
		[M+NH_4_]^+^	542.2241	525.1962 (2), 363.1442 (71), 345.1336 (100), 331.1180 (18), 225,225.0760 (7), 193.0499 (5), 165.0549 (9), 121.0650 (76)	−1.7	3.4		
42	21.46	[M-H]^−^	601.2122	601.2116 (8), 403.1241 (7), 223.0600 (4), 197.0813 (100), 179.560 (1), 153.0910 (24)	2.6	7.0	C_27_H_38_O_15_	2″-epi-frameroside/isomer
		[M+NH_4_]^+^	620.2547	441.1762 (39), 423.1654 (82), 391.1392 (25), 373.1291 (13), 331.1182 (100), 225.0762 (28), 199.0970 (37), 193.0499 (24), 165.0549 (50)	0.3	32.7		
43	22.07	[M+NH_4_]^+^	610.2504	593.2243 (1), 471.1508 (23), 447.1658 (7), 325.0927 (100), 309.0979 (7), 181.0.500 (3), 163.0394 (52)	−1.7	11.4	C_29_H_36_O_13_	caffeic acid derivative
44	22.89	[M-H]^−^	1071.3541	909.3030 (10), 839.2599 (42), 807.2696 (19), 685.2338 (63), 523.1813 (100), 453.1397 (49), 421.1494 (29), 403.1245 (28), 299.1135 (24), 223.0604 (35), 179.0558 (6)	2	8.8	C_48_H_64_O_27_	oleonuezhenide/isomer
45	23.04	[M+NH_4_]^+^	476.2498	476.2500 (37), 459.2234 (99), 271.0817 (100), 253.0711 (17), 171.1383 (58), 145.0499 (17), 127.0401 (15)	−1.6	9.3	C_22_H_34_O_10_	unidentified
46	23.46	[M+NH_4_]^+^	476.2496	476.2499 (19), 459.2233 (100), 271.0819 (91), 253.0714 (17), 171.1385 (91), 145.0499 (16), 127.0403 (14)	−1.2	6.9	C_22_H_34_O_10_	unidentified
47	23.73	[M+NH_4_]^+^	622.2498	587.2126 (5), 471.1506 (17), 441.1552 (12), 325.0927 (100), 309.0979 (10), 279.1023 (9), 181.1119 (1), 163.0394 (58), 117.0700 (16)	−0.5	4.4	C_30_H_36_O_13_	caffeic acid derivative
48	23.73	[M+NH_4_]^+^	594.2546	577.2293 (1), 455.1556 (25), 431.1711 (8), 309.0980 (100), 165.0551 (2), 147.0446 (29)	−0.2	11.5	C_29_H_36_O_12_	coumaric acid derivative
49	24.15	[M+NH_4_]^+^	680.2553	680.2553 (8), 541.1561 (13), 517.1714 (21), 395.0981 (100), 377.0878 (4), 165.0551 (4), 147.0445 (19)	−1.1	7.3	C_32_H_38_O_15_	coumaric acid derivative
50	25.10	[M-H]^−^	587.2124	587.2120 (100), 441.1748 (6), 307.1035 (7), 163.0391 (4), 145.0287 (44), 117.0342 (15)	1.7	13.8	C_30_H_36_O_12_	coumaric acid derivative

FA—formic acid; Hex—hexose; dHex—deoxyhexose; MaA—malonic acid.

**Table 2:** A comparison of the antioxidant activity of the extract from *S. vulgaris* flowers (10 µg/mL), and the extract from *T. officinalis* flowers (10 µg/mL) in human plasma treated with H_2_O_2_/Fe^2+^ (in vitro).

	The Extract from *S. vulgaris* Flowers	The Extract from *T. officinalis* Flowers
% of inhibition of lipid peroxidation	26.0 ± 15.1 (*p* < 0.05)	18.6 ± 7.9 (*p* < 0.05)
% of inhibition of protein carbonylation	27.7 ± 9.3 (*p* < 0.01)	28.9 ± 10.1 (*p* < 0.05)

## Data Availability

Dataset available on request from the authors.

## Abbreviations

The following abbreviations are used in this manuscript:

APTT	activated partial thromboplastin time
CUPRAC	cupric ion reducing antioxidant capacity
CVDs	cardiovascular diseases
DPPH•	2,2-diphenyl-picrylhydrazyl
FRAP	ferric-reducing antioxidant power
IC_50_	half maximal inhibitory concentration
MAP	mitogen-activated protein kinase
PT	prothrombin time
ROS	radical oxygen species
TBARS	thiobarbituric acid reactive substances
TT	thrombin time
